# An integrative review on unveiling the causes and effects of decision fatigue to develop a multi-domain conceptual framework

**DOI:** 10.3389/fcogn.2025.1719312

**Published:** 2026-01-09

**Authors:** Nurul Ahad Choudhury, Pratima Saravanan

**Affiliations:** School of Industrial Engineering and Management, Oklahoma State University, Stillwater, OK, United States

**Keywords:** cognitive depletion, conceptual framework, decision fatigue, decision-making, integrative review

## Abstract

Decision fatigue leads to reduced efficiency in the rate and quality of decisions. Thus, examining the reasons behind decision fatigue and comprehending its influence on decision-making in various professional domains is pivotal to improving the decision-making process. The aim of this integrative review is to investigate the causes and effects of decision fatigue from the existing literature and develop a framework that can be applied across different domains. A comprehensive literature search in three databases identified 1,027 articles on decision fatigue. After screening the articles using Integrative reviews and Meta-Analysis (PRISMA) and Joanna Briggs Institute (JBI) appraisal methods, 23 articles investigating decision fatigue across various domains were selected. The selected articles were investigated through root cause analysis and thematic synthesis. Findings revealed ten causes of decision fatigue, which were classified as individual, organizational, and external causes, and four primary effects and seven secondary effects of decision fatigue. Using these findings, a conceptual framework that offers a comprehensive understanding of decision fatigue across diverse domains was developed. Knowledge of what causes decision fatigue can help optimize the decision-making process for decision makers. This study contributes to the concept of decision fatigue within organizational settings to enhance organizational behavior, psychology, and offers implications for improving decision-making processes in diverse professional domains. The findings can help develop interventions to mitigate decision fatigue and improve overall decision-making.

## 1 Introduction

An average American adult makes approximately 35,000 decisions per day, comprising both personal and organization-level (work-related) decisions (Pignatiello et al., 2020). At both levels, decisions range from mundane individual responsibilities to critical strategic choices with high stakes and implications. The process of decision-making can be, at times natural and subconscious, whereas sometimes it involves conscious planning, hence requiring cognitive effort (Clay et al., 2022; Ang et al., 2023). Moreover, decision-making can be more challenging when it involves selecting the best option among several alternatives and efficiently deriving the desired purpose of the decision-making process (Lunenburg, 2010). Such complexities can lead to cognitive biases and errors, which are responsible for half of all the wrongful decisions within organizations (Nutt, 1999). The efficiency of the decision-making process diminishes as the complexity of decisions and the frequency of consecutive decision-making increase (Fernández-Miranda et al., 2023; Wisiecka et al., 2023). This phenomenon of reduced cognitive performance during successive decision-making under high workload is termed as “decision fatigue.”

Decision-making is a complex cognitive process, and any errors or biases can often lead to detrimental consequences, especially in high-risk domains (Lighthall and Vazquez-Guillamet, 2015; Benke et al., 2021). For instance, in healthcare, decision-making during clinical diagnosis is highly challenging as decisions are often made under pressure, thus leading to systematic errors (Croskerry and Nimmo, 2011; Restrepo et al., 2020; Trowbridge et al., 2020). Cognitive oversights or incomplete information can cause diagnostic errors and delays in treatment, worsening patient conditions (Thammasitboon and Cutrer, 2013; Barwise et al., 2021). Similarly, the aviation industry also operates in a dynamic and high-stakes environment where decision-making errors can have severe consequences. According to NASA, 80% of aviation accidents occur due to human errors in the decision-making process during uncertain circumstances (Kale et al., 2023). Therefore, slight deviations in rational thinking and judgment during decision-making can cause individuals to make less optimal decisions in many other professions, such as the judiciary (Malegiannaki et al., 2025), finance, and law (Larrick and Lawson, 2021).

Decision fatigue refers to the deteriorating quality of decisions made by an individual following a prolonged decision-making period (Pignatiello et al., 2020), and is a result of exhaustion, and impaired emotional and cognitive performance leading to irrational or impulsive decision-making (Baumeister et al., 1998; Stewart et al., 2012). The conceptual analysis of decision fatigue by Pignatiello et al. (2020) identified its antecedents, attributes, examples, and consequences, and recognized three primary antecedent themes (decisional, self-regulatory, and situational) and three attributional themes (behavioral, cognitive, and physiological). When these interconnected themes accumulate, decision-makers experience reduced motivation to exert cognitive effort, leading to decision avoidance and reduced efficiency and quality of decisions. For example, German car buyers who initially preferred customizing an Audi were more likely to accept the default options after going through a complex decision-making process during customization (Levav et al., 2010). The concept of decision fatigue was derived from a similar term called “ego depletion”, which is also based on the notion that cognitive resources can be depleted over time. Ego depletion is defined as the mental effort required for self-control in critical situations that exhausts cognitive resources, ultimately leading to decision fatigue (Baumeister and Vohs, 2007; Inzlicht and Schmeichel, 2012). However, the resource-based ego depletion model has been subject to considerable criticism over the years. Studies by Hagger et al. (2016) and Vohs et al. (2021) showed that several preregistered pragmatic tests failed to replicate the canonical ego depletion effect. Therefore, current concepts of ego depletion emphasize a process of motivational and attentional recollection rather than the depletion of resources (Cannon et al., 2019; Inzlicht and Schmeichel, 2012). The self-regulatory model of resource Scarcity by Cannon et al. (2019) states that individuals experiencing scarcity of cognitive resources usually respond through two distinct psychological routes: a scarcity-reduction route to reduce the discrepancy in resources, and a control-restoration route to reestablish diminished personal control. According to this framework, decision fatigue can be explained as a form of self-regulatory failure due to the scarcity of cognitive resources and a shift in attentional/motivational priorities that disrupts controlled decision-making. Collectively, these shifts from ego depletion theory suggest that decision fatigue emerges when individuals divert from effortful controlled decision-making to less effortful, erroneous decision-making under high cognitive demand and self-regulatory failure.

Several studies highlighted the negative effects of decision fatigue on the quality of decision-making (Persson et al., 2019; Zheng et al., 2020; Torres and Williams, 2022; Treasure et al., 2022). Frazier et al. (2022) highlighted that participants' perception, understanding, and prediction levels are affected by increasing decision fatigue. While basic perception remained stable, higher-order cognitive functions (understanding and prediction) declined significantly over time. Moreover, higher mental efforts are required to make complex decisions in high-stakes situations, such as critical clinical operations (Thompson et al., 2013; Grignoli et al., 2025), or emergency response scenarios like firefighting (Okoli and Watt, 2018; Choudhury and Saravanan, 2025a). Jia et al. (2022) investigated the effects of decision fatigue on risk preference and found that mentally fatigued individuals often preferred low-risk and low-return options rather than high-risk and high-return options. Moreover, decision fatigue reduces the performance of individuals in their workplace as they tend to make less optimal choices under fatigued conditions (Linder et al., 2014). These studies highlight the importance of further investigating this phenomenon of decision fatigue in an organizational setting. Hence, it is critical to investigate the causes of decision fatigue and understand its impact on decision-making across different occupational domains.

Existing literature has explored the origin of decision fatigue and how it influences an individual in specific domains such as healthcare, judiciary, and finance. However, research on generalizing these findings across multiple domains has been limited and inconclusive. For example, Andersson et al. (2025) conducted an empirical test of decision fatigue among healthcare professionals and found no evidence of decision fatigue. This finding further highlights the importance of exploring the causes and effects of decision fatigue while remaining sensitive to contextual boundary conditions within each domain. Moreover, notable heterogeneity has been observed in the antecedents and consequences across different domains (Pignatiello et al., 2020). For example, different organizational factors (e.g., responsibilities, duration of shifts) may cause decision fatigue differently in clinical and financial environments. However, aggregation into an integrative multi-domain framework is essential to understand the shared underlying cognitive factors that cause decision fatigue in individuals, regardless of the work domain. Identifying domain-specific causes and effects and integrating them into a single framework can help individuals across fields better understand how decision fatigue develops in their own domains. Thus, the overarching objective of this study is to investigate the causes and effects of decision fatigue from the existing literature and develop a multi-domain framework. The developed multi-domain framework integrates antecedent causes (i.e., individual, organizational, and external) with observed effects or consequences (i.e., primary and secondary effects) to extend the antecedent-attribute-consequence concept of decision fatigue to multiple organizational domains. A well-defined conceptual framework can aid practitioners, policymakers, and researchers in improving the effectiveness and accuracy of organizational decision-making by making them aware of the causes and effects of decision fatigue beforehand.

## 2 Materials and methods

We employed an integrative review-based approach (Whittemore and Knafl, 2005; Cronin and George, 2023) to develop a conceptual framework for decision fatigue. Integrative reviews strive to synthesize past research relevant to a specific inquiry, aiming to provide an integrated understanding of the inquired topic from the fragmented previous findings (Torraco, 2005). Thus, this study allows for integrating diverse findings and identifying common themes across the past literature to develop a conceptual framework.

### 2.1 Search strategy and selection criteria

The integrative review followed the Preferred Reporting Items for Systematic reviews and Meta-Analysis (PRISMA) guidelines primarily to ensure transparency of the search strategy, screening, and inclusion process. After consulting with a librarian, we conducted a thorough literature search using relevant keywords, MeSH terms, and Boolean operators. To identify all relevant articles, we used “cognitive fatigue,” “compassion fatigue,” and “alert fatigue” as MeSH terms for “decision fatigue.” Articles retrieved from these terms were screened manually, and studies focusing solely on these distinct constructs were excluded unless they explicitly addressed decision fatigue. A thorough literature search across Scopus, APA PsycInfo, PubMed, and a bibliometric search resulted in 1,027 articles. The full search strings for the main terms for the three databases are shown in Appendix A. Only the studies that primarily discussed decision fatigue and/or the performance decline among individuals who went through the process of extensive and successive decision-making were included in this review. Moreover, the following exclusion criteria were used. (1) Studies that focused solely on adjacent topics (e.g., physical fatigue, compassion fatigue, alert fatigue, general cognitive workload) without directly linking them to decision fatigue were excluded. (2) Literature review and conceptual analysis articles, and other articles that were irrelevant to the research objectives were excluded. (3) Peer-reviewed journal articles written in a non-English language and published before 2001 were excluded. There were no restrictions on the domain or demographics, as the research is aimed at analyzing the impact of decision fatigue and is not confined to any specific domains or regions. Abstracts and full-text screening were conducted independently by two reviewers, and discrepancies were resolved through discussion until full consensus was achieved, using an open discussion strategy (Chinh et al., 2019). Details of the search strategy and inclusion/exclusion criteria are preregistered and the anonymous link is: https://osf.io/e59fh/?view_only=cb011414f1b84f49adf5dc1d1a19b581. This repository serves as a data and materials archive, rather than a preregistration record.

### 2.2 Quality assessment

The qualitative Joanna Briggs Institute (JBI) Quality Appraisal tool (Santos et al., 2018; Aromataris and Munn, 2020), a process used to assess the methodological rigor and validity of research studies, was used to evaluate the quality of the articles. The purpose of quality appraisal is to evaluate the congruity and quality of the method and findings in a study. The appraisal method includes 10 questions to evaluate the design, bias, confounding, and data analysis of the articles (Munn et al., 2020). For each question, a “positive,” “negative,” “unsure,” or “not applicable” rating is assigned after carefully reviewing the studies. Two reviewers independently completed the appraisals, and discrepancies were resolved through discussion until consensus was achieved. If any article was deemed substandard by the reviewers, it was excluded from the review.

### 2.3 Data extraction

The selected articles were thoroughly synthesized through a data extraction process to find specific themes related to the causes and effects of decision fatigue. For data synthesis, each article was read by two reviewers (a doctoral student and a faculty member, both experienced in qualitative research) multiple times, and the relevant data were extracted. We performed thematic coding on the 23 articles to generate themes and develop a conceptual framework, following the six-step thematic analysis process designed for conceptual model development outlined by Naeem et al. (2023). The findings from the extracted 23 articles were qualitatively coded through iterative and axial coding (Braun and Clarke, 2006) to identify 10 causes and four primary effects of decision fatigue. The thematic categories (i.e., causes, primary and secondary effects) were derived through iterative comparison of extracted findings and verified through full consensus discussion among the authors.

## 3 Results

**Figure 1:**
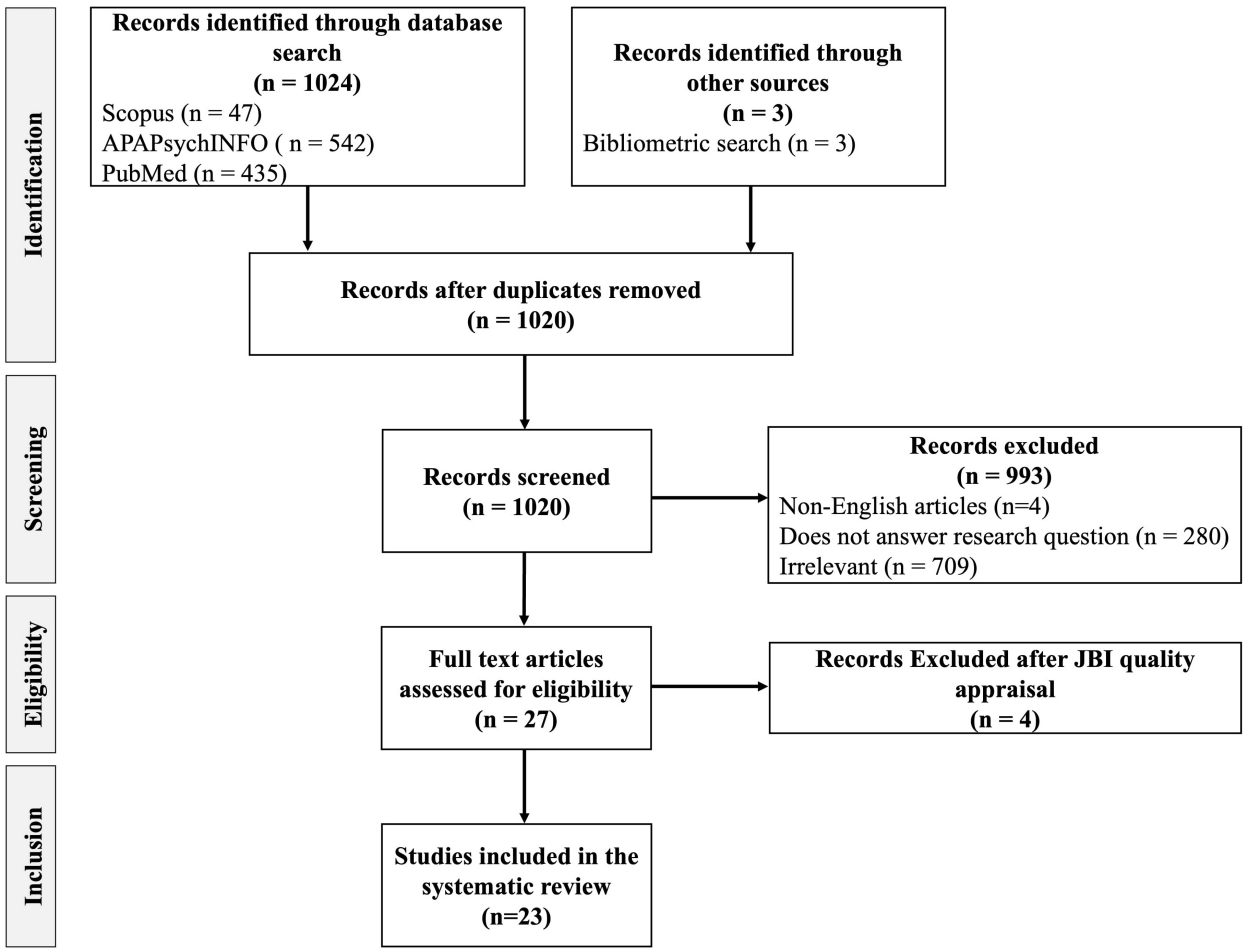
PRISMA flowchart of the integrative review process. Flow diagram showing the integrative review selection process. From 1,024 records identified through Scopus (47), APA PsycINFO (542), and PubMed (435), plus 3 additional records from bibliometric search, 1,020 records remained after duplicates were removed. After screening, 993 records were excluded, including 4 non-English, 280 unrelated to the research question, and 709 irrelevant studies. Twenty-seven full-text articles were assessed for eligibility; 4 were excluded after JBI quality appraisal, resulting in 23 studies included in the final review.

Out of the 1,027 articles identified in the initial database search, 1,020 articles remained after removing duplicates (see Figure 1). These records were screened according to the inclusion/exclusion criteria. Eventually, 997 out of the 1020 articles were excluded, and the remaining 27 articles were assessed for eligibility using the JBI appraisal tool. Four articles were excluded from consideration because of low scores. The details of the JBI appraisal process are presented in Table 1. The remaining 23 articles that met all the criteria were included in the integrative review.

**Table 1:** Risk of bias for included studies using Joanna Briggs Institute criteria.

**Authors (year)**	**Is there congruity between the started philosophical perspective and the research methodology?**	**Is there congruity between the research methodology and the research question or objectives?**	**Is there congruity between the research methodology and the methods used to collect data?**	**Is there congruity between the research methodology and the representation and analysis of data?**	**Is there congruity between the research methodology and the interpretation of results?**	**Is there a statement locating the researcher culturally or theoretically?**	**Is the influence of the researcher on the research, and vice-versa, addressed?**	**Are participants, and their voices, adequately represented?**	**Is the research ethical according to current criteria or, for recent studies, and is there evidence of ethical approval by an appropriate body?**	**Do the conclusions drawn in the research report flow from the analysis, or interpretation of the data?**	**Total score**
(Dong et al. 2024)	+	+	+	+	+	+	–	+	–	+	8
(Jiao 2024)	+	+	+	+	+	+	+	+	U	+	9
(Rashid 2024)	+	U	+	+	+	+	+	+	–	+	8
Fernández-Miranda et al. (2023)	+	+	+	+	+	U	U	+	+	+	8
Treasure et al. (2022)	+	+	+	+	+	U	+	+	+	+	9
Torres and Williams (2022)	+	+	+	+	+	+	–	+	–	+	8
Shroff and Vamvourellis (2022)	+	+	+	+	+	+	–	+	–	+	8
Natal and Saltzman (2022)	+	+	+	+	+	+	+	+	+	+	10
Angoff et al. (2022)	+	+	+	+	+	+	U	+	+	+	9
Hunt et al. (2021)	+	U	+	+	+	+	+	+	+	+	9
Trinh et al. (2021)	+	+	+	+	+	+	+	+	+	+	10
Allan et al. (2019)	U	+	+	+	+	–	+	+	+	+	8
Ma et al. (2021)	+	+	+	+	+	+	+	+	U	+	9
Baer and Schnall (2021)	+	U	+	+	+	+	+	+	–	+	8
Hughes et al. (2020)	+	U	+	+	U	U	+	–	+	–	5
Zheng et al. (2020)	+	U	+	+	U	U	+	+	+	+	7
Persson et al. (2019)	+	+	+	+	+	+	+	+	–	+	9
Hirshleifer et al. (2019)	+	U	+	+	+	+	+	+	+	+	9
Pignatiello and Hickman (2019)	+	+	+	+	+	+	+	+	+	+	10
Massar et al. (2018)	U	+	U	–	+	+	U	U	–	+	4
Olsen et al. (2017)	+	+	U	+	+	+	+	+	–	+	8
Polman and Vohs (2016)	U	–	+	+	+	+	+	+	–	+	7
Mullette-Gillman et al. (2015)	+	+	+	+	+	+	+	+	+	+	10
Dragone (2009)	U	U	+	+	+	U	+	+	–	+	6
Simioni et al. (2009)	+	U	U	+	+	+	+	U	–	+	6
Linder et al. (2014)	+	+	+	+	+	+	+	+	+	+	10
Vohs et al. (2008)	+	U	+	+	+	U	+	+	U	+	7

+ = yes; – = no; U, unsure; NA, not applicable.

### 3.1 Study characteristics

We reviewed 23 articles on decision fatigue to identify its causes and effects. The articles included in the review focused on investigating decision fatigue in different organizational settings, with *n* = 13 articles in healthcare, *n* = 5 articles in financial (economic) analysis, *n* = 2 in the judiciary system, *n* = 1 in academic librarianship, *n* = 1 in parenting practices, and *n* = 1 in multiple organizations. Table 2 summarizes the 23 articles included in this review.

**Table 2:** Description of included studies.

**Authors (year)**	**Domain**	**Sample size/setting**	**Analysis methods/tools**	**Outcomes measured**	**Causes of decision fatigue**	**Effects of decision fatigue**	**Major findings**
Dong et al. (2024)	Healthcare	Frontline nurses (*n* = 14)	Interpretative phenomenological analysis (IPA)	Cognition, influence, and attitude toward decision fatigue; factors leading to and mitigating decision fatigue	An imperfect system, internal environment constraint	No significant effect reported	Many nurses were unaware of decision fatigue, decision fatigue increased as shifts progressed, identified avoidant factors of decision fatigue
Jiao (2024)	Financial analysis	Analysts' earnings forecasts (*n* = 605,835)	Regression analysis	Forecast accuracy, order of forecast issuance, and effects of decision fatigue	High number of forecasts issued in a day (high workload)	Reduced forecast accuracy, increased reliance on heuristic forecasting	Decision fatigue negatively affects forecast accuracy analysts issue forecasts for important firms earlier in the day to avoid decision fatigue
Rashid (2024)	Multiple domains	Executive professionals (*n* = 373)	Correlation analysis, moderated-mediation model, Pearson correlation, regression analysis	Relationship between decision fatigue, workplace well-being, pragmatic prospection, and organizational culture	Cognitive overload, weak organizational culture	Decreased workplace well-being, subdued performance levels	Decision fatigue negatively affects workplace well-being, reducing employee satisfaction and performance.
(Fernández-Miranda et al. 2023)	Healthcare	Healthcare workers (*n* = 856); 66.2% women, 33.8% men; specialists (25.07%), general practitioners (9.43%), residents (2.02%), nurses (8.49%), other professionals (10.11%), technician (9.84%), student (1.62%)	Statistical analysis (correlation)	Levels of compassion and decision fatigue during COVID-19	Duration of the decision-making period, responsibility, and complexity involved in the decision-making	Erroneous decisions	Decision fatigue was lower for specialists, general practitioners, and technicians compared to those working at private hospitals
(Treasure et al. 2022)	Healthcare emergency department	Patients (*n* = 9,724)	Retrospective cohort study (chi-square test and *t*-test)	Admission rate and throughput metrics in relation to time	Duration of the decision-making period, frequency of decisions	Ineffective decision-making	Decision fatigue caused less efficient patient flow in the later part of the shift
(Torres and Williams 2022)	Judiciary	Judiciary cases (*n* = 284); 157 decided by judge 1 in 18 sessions, 127 by judge 2 in 16 sessions	Statistical analysis (multivariate regression)	Effect of decision fatigue on engagement, judicial deviations, bail amount	Duration of the decision-making period, frequency of decisions	Ineffective decision-making, erroneous decisions	Decision fatigue caused by the case order and session duration limited the engagement for one judge and affected set bail amounts for both judges
(Shroff and Vamvourellis 2022)	Judiciary	Judiciary cases (*n* =39,157)	Statistical analysis (covariate analysis)	How judicial release and bail decisions are influenced by the time an arraignment occurs.	Duration of the decision-making period, impact of time, and availability of breaks	Ineffective decision-making, erroneous decisions	Decision fatigue influences pretrial release judgments, although effects are small and inconsistent
(Natal and Saltzman 2022)	Academic librarianship	Academic librarians (*n* = 85,752); conducted through an online 29-question survey, 353 valid responses	Quantitative and qualitative survey study	Effect of various factors on decision making	Duration of the decision-making Period, Frequency of decisions	Ineffective decision-making	Librarians may select the easiest option if they are faced with a lot of options when fatigued thus affecting the decision-making process
Angoff et al. (2022)	Parenting practices	Parents (*n* = 140); 88.57% female; 88.41% white; 87.59% married	Qualitative and quantitative analysis with survey	Changes in parenting behavior due to decision fatigue and stress during COVID-19	Uncertainty	Ineffective decision-making	Parents get more vulnerable to stress on parenting behaviors related to food and physical activity when they are decision fatigued
Hunt et al. (2021)	Healthcare	Patient appointments (*n* = 15,81,826); median (IQR) age at 54 (37–66) years, with 54% under 55	Retrospective cohort study	Association of appointment time with PSA testing likelihood	Duration of the decision-making period	Ineffective decision-making	Results show a decline in clinicians' likelihood of ordering PSA tests (decision-making) when fatigued cognitively
Trinh et al. (2021)	Healthcare	Patients (*n* = 3,342); 2,013 (55%) female in the first hour and 1,329 (61% female) in the last hour	Two sample *t*-tests, the Wilcoxon test, Kruskal–Wallis test, and multivariate linear regression	No. of diagnosis tests ordered, and no. of diagnoses assessed per patient visit over first vs. last hour	Duration of the decision-making period	Ineffective decision-making	Results showed the prevalence of time-of-day effects on clinician decision-making, particularly on the number of diagnostic tests ordered per patient
Allan et al. (2019)	Healthcare	Nurses (*n* = 150); mean age =44 years (SD = 7.5); female (*n* = 142)	Mixed effect logistic regression	A measure of shift from effortful to a more conservative decision since the last break increases	Impact of time and availability of breaks	Conservativeness in decision-making	Nurses become less efficient in decision-making as time since break and work demand increases
(Ma et al. 2021)	Economic analysis	Institutions (*n* = 1,866)	Statistical analysis	Effect of Decision Fatigue on institutional bidding behavior	Frequency of decisions, Order of the decisions	Ineffective decision-making	Institutions' bid accuracy declined as the number of previous decisions made increased in a day
Baer and Schnall (2021)	Economic analysis (finance)	Credit loan applications (*n* = 26,501)	Logistic regression analysis	Quantification of the cost of decision fatigue		Ineffective decision-making	Credit loan approvals across the course of the day decreased during midday compared with early or later in the workday, highlighting the presence of decision fatigue
Zheng et al. (2020)	Healthcare emergency department	Patients (*n* = 87,752); 52.4% female, mean age of 62.4	Random effects regression	Change in decision fatigue over time	Duration of the decision-making period	Ineffective decision-making	The rates of CT head and abdomen and ED LOS decreased as the shift progressed
Persson et al. (2019)	Healthcare	Patients (*n* = 848); surgery department (*n* = 270), non-surgery department (*n* = 573)	Logistic regression analysis	Effect of decision fatigue on the decision of doing an operation or not	Duration of the decision-making period	Ineffective decision-making	Surgeons are less likely to schedule an operation for a patient toward the end of their work shift
Hirshleifer et al. (2019)	Economic analysis	Forecasts from the I/B/E/S database (*n* = 386,924)	Regression models, fixed effects, and statistical analysis of analyst forecasts	Forecast accuracy, heuristic decision-making, and stock market reaction	Number of forecasts and number of analyst days (high workload)	Decline in forecast accuracy. Increased reliance on heuristics (herding)	Analysts' forecast accuracy reduces as they issue more forecasts in a day, fatigued analysts depend more on heuristic decision-making
Pignatiello and Hickman (2019)	Healthcare	Surrogate decision makers (*n* = 97); female (72%) and white (75%).	Multiple regression analysis	Association of cognitive regulation and decision fatigue on cognitive load	Frequency of decisions	Perceived complexity in decision-making	Decision fatigue, age, and anxiety were significant predictors of cognitive load, indicating the association of decision fatigue with increased intrinsic cognitive load
Olsen et al. (2017)	Healthcare/food choice	Survey participants (*n* = 1431), complete responses (*n* = 891)	Empirically stated choice survey	Effect of time of day on error variance in the food choices	Duration of the decision-making period	Erroneous decisions	Results show evidence of time-of-day effect on decision-making in stated food choices as well as estimated market share predictions
Polman and Vohs (2016)	Healthcare/random participants	Random participants (*n* = 957)	Statistical hypothesis testing (chi-square test)	Investigated the occurrence of decision fatigue of decisions for others compared to self	Presence of different alternatives of decisions	Ineffective Decision-making	Results highlight that making decisions for others is less depleting than making choices for oneself
Mullette-Gillman et al. (2015)	Economic analysis	University students (*n* = 72); control group (*n* = 25) and fatigued group (*n* = 47)	Between-subjects manipulation design	Effect of decision fatigue on economic decision-making	Presence of different alternatives of decisions	Perceived complexity in the decision-making	Decision fatigue destabilizes economic decision-making, resulting in erratic preferences and informational strategies that reduce decision quality
Linder et al. (2014)	Healthcare	Acute respiratory infection (ARI) visits by adults (*n* = 21,867) aged 18–64 years, across 204 clinicians in 23 primary care practices	Logistic regression models with fixed effects for clinicians	Likelihood of antibiotic prescribing over time in clinic sessions	Time of the day, cognitive load accumulation throughout the day	Antibiotic prescribing increased throughout the day, higher odds ratios for prescriptions in later hours	Antibiotic prescribing increased over shift hours, with adjusted odds ratios rising from 1.01 in the second hour to 1.26 in the fourth hour, while decision fatigue disrupted clinicians' resistance to prescribing unnecessary antibiotics
Vohs et al. (2008)	Healthcare/random participants	Random participants (*n* = 34)	Statistical analysis	Compared decision fatigue for making choices between self and others	Uncertainty	Self-control in terms of quality of decision making	Making choices is more difficult than implementing choices made by someone else as it reduces the quality and quantity of decision-making

### 3.2 Causes of decision fatigue

The synthesis of findings from the 23 articles revealed ten causes of decision fatigue: (1) duration of decisions (time of the day), (2) responsibilities involved with the decision, (3) complexity of decision-making, (4) availability of breaks, (5) high workload, (6) weak organizational culture, (7) presence of alternative decisions, (8) frequency of decision-making, (9) order of decisions, and (10) uncertainty of decisions. After synthesizing the results, we classified the causes into 3 major categories, (1) organizational causes, (2) individual causes, and (3) external causes. The causes were derived from the qualitative codes generated through the thematic coding process, followed by clustering the codes into the analytical themes (causes). For example, the analytical theme of the cause “duration of decisions (time of the day)” was derived from multiple codes from the findings from the extracted article, such as time of day Zheng et al., (2020), time of shift Treasure et al., (2022), time period of decision-making Shroff and Vamvourellis, (2022). These codes were first grouped into a common descriptive theme, “effect of time of decision-making session or period,” which, after discussion and consensus among the authors, was synthesized into the final analytical theme. A similar thematic analysis was conducted to identify other causes of decision fatigue across the 23 identified articles.

#### 3.2.1 Organizational causes

Several causes of decision fatigue were grouped under organizational causes. Organizational causes arise from demanding job responsibilities, task structures, job complexity and institutional constraints Ganster, (2005); Phillips-Wren and Adya, (2020), which initiate cognitive Alyahya et al., (2021); Dong et al., (2024), physical Gander et al., (2011), and organizational inefficiency Lin and Orvis, (2016). The identified organizational causes are—the duration of decisions, responsibilities involved with the decisions, the complexity of decision-making, availability of breaks, high workload, and weak organizational culture.

***Duration of Decisions (Time of the Day)*. **Most of the researchers have associated the prolonged decision-making period to decision fatigue as an influential reason in workplaces Linder et al., (2014); Olsen et al., (2017); Zheng et al., (2020); Baer and Schnall, (2021); Hunt et al., (2021); Trinh et al., (2021); Natal and Saltzman, (2022); Shroff and Vamvourellis, (2022); Torres and Williams, (2022); Treasure et al., (2022); Fernández-Miranda et al., (2023). Time of the day/period is frequently associated with decision fatigue, as decisions made during the later parts of the day/period tend to be affected by decision fatigue. Regardless of the domains, past studies have identified that individuals make better decisions in the first half of the day compared to the second half of the day. Trinh et al. (2021) showed that some physicians ordered significantly different numbers of diagnostic tests and patient diagnoses in the first hour vs. the last hour of the shift (*p* ≤ 0.04). Torres and Williams (2022) investigated the effect of the duration of the decision-making period on the judicial decision-making process of two judges. The results indicated that the order of case presentation during the day influenced judicial decision-making, with cases presented in the latter half of the day having a lower level of engagement and lower bail amounts. These studies indicate that time has a substantial effect on causing decision fatigue and on the overall effectiveness of the decision-making process. However, Shroff and Vamvourellis (2022) found a negligible effect of time on judicial release and bail decisions in pretrial arrangements. Whereas, Zheng et al. (2020) did not find any effect of the time of the shift on the decision-making of emergency physicians over an eight-hour shift, which indicates the possibilities of other moderating causes that need to be explored in future.

***Complexity of Decision-Making*. **Complex and challenging tasks can cause decision fatigue, since specific activities in an organization are more complicated than other routine activities. For example, emergency departments in healthcare have patients with severe health conditions, and their treatment requires intricate diagnoses and surgeries compared to patients in the general ward Zheng et al., (2020). Similarly, Fernández-Miranda et al. (2023) showed that the depletion of cognitive resources occurs sooner as the complexity of activities increases with the change in the role of professionals, hence leading to increased decision fatigue.

***Responsibilities Involved with Decision*. **Decision fatigue can often be caused by the weight of the responsibilities associated with the job. The findings from three articles (Vohs et al., 2008; Natal and Saltzman, 2022; Fernández-Miranda et al., 2023) indicate that decision fatigue can fluctuate depending on the diverse responsibilities held by an individual. Additionally, specific decisions may impose a greater cognitive effort and burden than others, and the importance of decision-making can differ among various professionals within a workplace. In essence, the complexity and nature of responsibilities and individual differences in the hierarchy of an organization contribute to the variability in decision fatigue. Fernández-Miranda et al. (2023) found that the extent of decision fatigue varied with the job responsibilities of healthcare workers during the COVID-19 pandemic. Decision fatigue was lower for specialists, general practitioners, and technicians in government hospitals and higher for those working at private hospitals because of the specific protocols maintained by the government hospitals for the physicians regarding patients' treatment. Moreover, decision fatigue also varies while making decisions for oneself compared to making decisions for others (Vohs et al., 2008; Natal and Saltzman, 2022), as making choices for others depletes cognitive resources faster and causes higher decision fatigue. In contrast, Polman and Vohs (2016) found that choosing for others may deplete fewer cognitive resources and result in lesser decision fatigue.

***Availability of Breaks*. **Four out of the 23 articles highlighted that the timing and number of breaks during a shift significantly impact decision fatigue. Decision fatigue can occur when individuals are required to make a series of choices without adequate rest or recovery. Allan et al. (2019) showed that the timing of breaks can have a crucial effect on the performance of nurses. The decisions made by nurses tend to become progressively more conservative and less efficient with each subsequent decision following their last break. Moreover, a food break or any other kind of meal break has a positive effect on relieving fatigue (Natal and Saltzman, 2022; Shroff and Vamvourellis, 2022). The presence of a break can rejuvenate the performance compared to the individual's performance prior to the break. Baer and Schnall (2021) found that approval rates for credit risk applications declined toward the middle of the workday and increased later in the afternoon (post lunch), indicating the effect of breaks during the shift. On the other hand, Shroff and Vamvourellis (2022) observed a negligible effect of breaks on decision fatigue in a study involving the decision-making process of judges in the judiciary system. Pretrial release rates remained unchanged after a meal break.

***High Workload*. **A high workload is associated with decision fatigue by depleting cognitive resources, thus forcing individuals to make numerous complex decisions in a limited time (Hirshleifer et al., 2019; Natal and Saltzman, 2022; Treasure et al., 2022). For example, Hirshleifer et al. (2019) identified workload as a cause of decision fatigue in financial analysts. Specifically, this study highlights that issuing multiple forecasts in a single day reduces decision quality, leading to increased reliance on heuristic decision-making. Moreover, Natal and Saltzman (2022) demonstrated how balancing multiple responsibilities and having to make repeated decisions can be overwhelming for librarians. Treasure et al. (2022) also showed that decision fatigue accumulates due to high patient volume rather than just shift length in the emergency medical department.

***Weak organizational culture*. **A highly disorganized and rigid organizational culture causes decision fatigue by increasing stress and cognitive overload. For instance, Rashid (2024) found that organizational culture moderates the relationship between decision fatigue and workplace well-being, pointing out that a rigid culture worsens fatigue effects. Moreover, Dong et al. (2024) highlighted that hospital policies and lack of institutional support led nurses to get stressed and prevented them from seeking help when fatigued.

#### 3.2.2 Individual causes

The deterioration in the quality of decision-making when a person undergoes repetitive decision-making is influenced by individual causes. These individual causes can be collectively defined as cognitive or emotional states within the decision maker, reflecting internal vulnerability within the individual (Stewart et al., 2012; Polman and Vohs, 2016). Individuals react differently in different situations and have unique abilities to cope with these situations, which can lead to variability in the amount of decision fatigue. These causes vary among individuals, regardless of their occupation. Three individual factors are identified in this literature review as causes of decision fatigue—presence of alternative decisions, frequency of decision-making, and order of decisions.

***Presence of Alternative Decisions*. **Decision-makers often seek options to select the best alternative at both organizational and individual levels. While having multiple options can be advantageous, the presence of multiple alternatives can also cause decision fatigue, as evaluating the alternatives becomes mentally taxing and time-consuming (Mullette-Gillman et al., 2015; Polman and Vohs, 2016). A survey-based study on librarians' decision-making process revealed that decision fatigue increased when multiple choices were involved, and some found decision-making to be more cognitively taxing and unpleasant because of the number of choices they had to consider. This suggests that having different alternatives can cause higher decision fatigue (Natal and Saltzman, 2022). Hence, selecting among multiple alternatives is more exhausting than simply thinking, comprehending, and developing singular choices.

***Frequency of Decision-making*. **Making multiple decisions in a limited time tends to exhaust the decision-making or thinking capabilities of an individual. This frequent successive decision-making not only depletes mental resources but also reduces the capacity for self-control, ultimately leading to a decline in decision-making quality (Polman and Vohs, 2016). Ma et al. (2021) showed the exact kind of depletion of mental resources and its detrimental effect on decision-making due to frequent decision-making in economic bidding analysis. This article observed the effects of decision fatigue on institutional bidding accuracy and revealed that bid accuracy decreased as the organizations made multiple consecutive decisions. This highlights the need for caution when it comes to the frequency of decision-making, as it can significantly impact the quality of decisions.

***Order of Decisions*. **The sequence or order of the decisions can also dictate decision fatigue. It is easier to make decisions during the initial phases of a task rather than the later phases, as the cognitive resources of the decision-maker start to deplete toward the end of the task. Torres and Williams (2022) investigated the effect of the order of decisions on decision fatigue in the judiciary system. Results showed that the order of the cases being presented and the session duration (first half or second half of the day) reduced decision-making quality by reducing engagement and lowering bail amounts for the two judges under observation.

#### 3.2.3 External causes

External factors can also substantially impact decision fatigue. External causes can be collectively defined as factors beyond individual and organizational causes (e.g., emergencies, environmental uncertainty) that can increase cognitive demands during decision-making (Johnson and Busemeyer, 2010; Phillips-Wren and Adya, 2020). Recognizing and dealing with these external factors is crucial for organizations and individuals seeking to reduce decision fatigue. Uncertainty about events and situations is one such external factor that can cause decision fatigue.

***Uncertainty of Decisions*. **Unforeseeable and uncertain external conditions can contribute to an increase in decision fatigue. Uncertainty in situations requires professionals to make rapid adjustments in decision-making, resulting in mental exhaustion and decision fatigue. People tend to experience mental exhaustion in challenging situations characterized by risk and ambiguity, leading to mental fatigue (Mullette-Gillman et al., 2015). Moreover, a study conducted by Angoff et al. (2022) found evidence of stress and decision fatigue in parenting practices related to food and physical activity during the uncertain events of the COVID-19 pandemic. The results show that perceived decision fatigue increased among parents during the pandemic. Other studies have also shown that decision fatigue can escalate during high-risk and uncertain situations, such as the outbreak of the COVID-19 pandemic (Fernández-Miranda et al., 2023).

### 3.3 Effects of decision fatigue

Decision fatigue can have several effects on individuals, influencing various aspects of their cognitive functioning and occupational performance. Individuals and organizations can foster better decision-making and increase performance and productivity by being aware of how decision fatigue impacts performance and taking proactive measures. All the articles in this integrative review have focused on one or more effects of decision fatigue. Table 1 presents the effects of decision fatigue analyzed in each article, which were later grouped into the four primary effects.

### 3.3.1 Primary effects

Primary effects are the immediate impacts on the performance of an individual under the influence of decision fatigue. Furthermore, secondary effects arise due to the primary effects of decision fatigue, further impacting an individual's performance and decision-making quality. For example, Allan et al. (2019) reported that nurses become increasingly likely to make conservative decisions as the time since their last rest break and the number of decisions made increase. This excerpt was first coded as a “conservative choice tendency.” When comparing similar codes across studies, this code was grouped under the broader primary effect category of “conservativeness in decision-making.” Furthermore, these conservative decisions resulted in reduced efficiency in decision-making and a slower rate of decisions, which were the identified secondary effects from this article. Thus, four primary effects and seven secondary effects of decision fatigue were identified across all 23 articles. The primary effects are (1) ineffective decision-making, (2) conservativeness in decision-making, (3) erroneous decisions, and (4) perceived complexity in decision-making.

***Ineffective Decision-making*. **Decision fatigue impedes the effectiveness of the decision-making process both at the individual and organizational levels. Six of the 23 articles indicated ineffective decision-making as the primitive effect of decision fatigue. As decision fatigue occurs in a person, the capacity to make efficient and logical decisions declines. Individuals might opt for more accessible, less effective choices or avoid making decisions altogether. Decision fatigue reduces the efficiency of decision-making when the performance deteriorates at the end of the shift or when several decisions are already taken (Torres and Williams, 2022; Treasure et al., 2022). Treasure et al. (2022) found that when physicians get fatigued, they fail to recognize the severity of the patients' condition and admit fewer patients at the end of their shifts. This study also highlighted that physicians require higher mental effort to make decisions toward the end of their shift. Other articles have shown that ineffectiveness in decision-making is caused by a declining rate of decision-making (Zheng et al., 2020; Hunt et al., 2021) and inconsistency in decision-making (Vohs et al., 2008; Persson et al., 2019). Decision fatigue often causes wrong or less efficient decisions to be made. Hunt et al. (2021) found that clinicians ordered fewer clinical tests as the day progressed, although prescribing the test would have been both appropriate and cost-effective for the patients. Another study showed that decision fatigue led to inconsistency in decision-making and caused a 10.5% reduction in the odds of surgery in surgeons' clinical decision-making (Persson et al., 2019). Furthermore, Hirshleifer et al. (2019) found that financial analysts who issue multiple forecasts in a single day exhibited lower forecast accuracy. As analysts became more mentally exhausted, they increasingly relied on heuristic decision-making strategies, which resulted in less accurate forecasts. Similarly, Jiao (2024) demonstrated that decision fatigue among analysts leads to forecast errors during complex financial predictions. Thus, decision fatigue impacts the effectiveness of an individual's decision-making by causing inefficiency and inconsistency and demanding higher effort in decision-making.

***Conservativeness in Decision-making***. Decision fatigue originates when a person is mandated to choose among various alternatives. We have discussed in Section 3.2.2 that choosing between different alternatives leads to decision fatigue. Decision-makers may feel overwhelmed and mentally exhausted due to making lots of decisions or numerous choices throughout the day. Decision-making becomes difficult in such situations, and individuals tend to make conservative decisions (Linder et al., 2014; Polman and Vohs, 2016; Allan et al., 2019; Baer and Schnall, 2021; Natal and Saltzman, 2022; Torres and Williams, 2022). Moreover, decision fatigue reduces the ability of an individual to make sound and well-sorted decisions. As a result, decision-making becomes complex and an individual might choose the safest option in a prolonged period of decision-making or when faced with many choices (Torres and Williams, 2022). For instance, Linder et al. (2014) observed this effect in healthcare, where fatigued physicians became more likely to prescribe antibiotics rather than engage in complex diagnostic reasoning as the working shift progressed. Moreover, Natal and Saltzman (2022) showed that librarians take the most straightforward option when subjected to multiple choices. Such practices of easy or suboptimal risk decisions were also observed in the finance sector because of decision fatigue (Baer and Schnall, 2021).

***Erroneous Decisions*. **As decision fatigue occurs from repetitive decisions, individuals become prone to making mistakes in evaluating and selecting the best alternative. Hence, decision fatigue can give rise to various adverse effects during decision-making and elevate the probability of errors. Five of the articles have highlighted that being aware of the potential consequences of decision fatigue can help individuals make more deliberate and thoughtful choices, reducing the likelihood of mistakes (Vohs et al., 2008; Olsen et al., 2017; Ma et al., 2021; Trinh et al., 2021; Shroff and Vamvourellis, 2022). Furthermore, Trinh et al. (2021) found differences in the number of diagnostic tests and the average number of diagnoses in the first vs. the last hour of a shift. This discrepancy was caused by decision fatigue arising from the difference in the time of the shift. The evidence of error in decision-making was also found in a food choice experiment as respondents provided less consistent answers during the afternoon than at other times of the day (Olsen et al., 2017). So, these articles emphasize that Decision fatigue may lead to errors in decision-making by reducing the rate of decision-making and by prompting an individual to make wrong and inconsistent decisions.

***Perceived Complexity in Decision-making*. **As individuals make decisions throughout the day, they utilize mental energy and engage in complex cognitive processes. This requires a higher cognitive load and eventually makes decision-making difficult and the decision makers start to perceive those decisions as highly complex ones (Mullette-Gillman et al., 2015; Pignatiello and Hickman, 2019; Torres and Williams, 2022). Decision fatigue was found to be a reason for higher intrinsic cognitive load in surrogate decision-makers of critically ill patients (Pignatiello and Hickman, 2019). The findings showed that decision fatigue caused individuals to reach a depleted state of mental resources and increased intrinsic cognitive load. Thus, decision fatigue causes complexity in the decision-making process and demands higher mental effort from the decision-maker.

**Figure 2:**
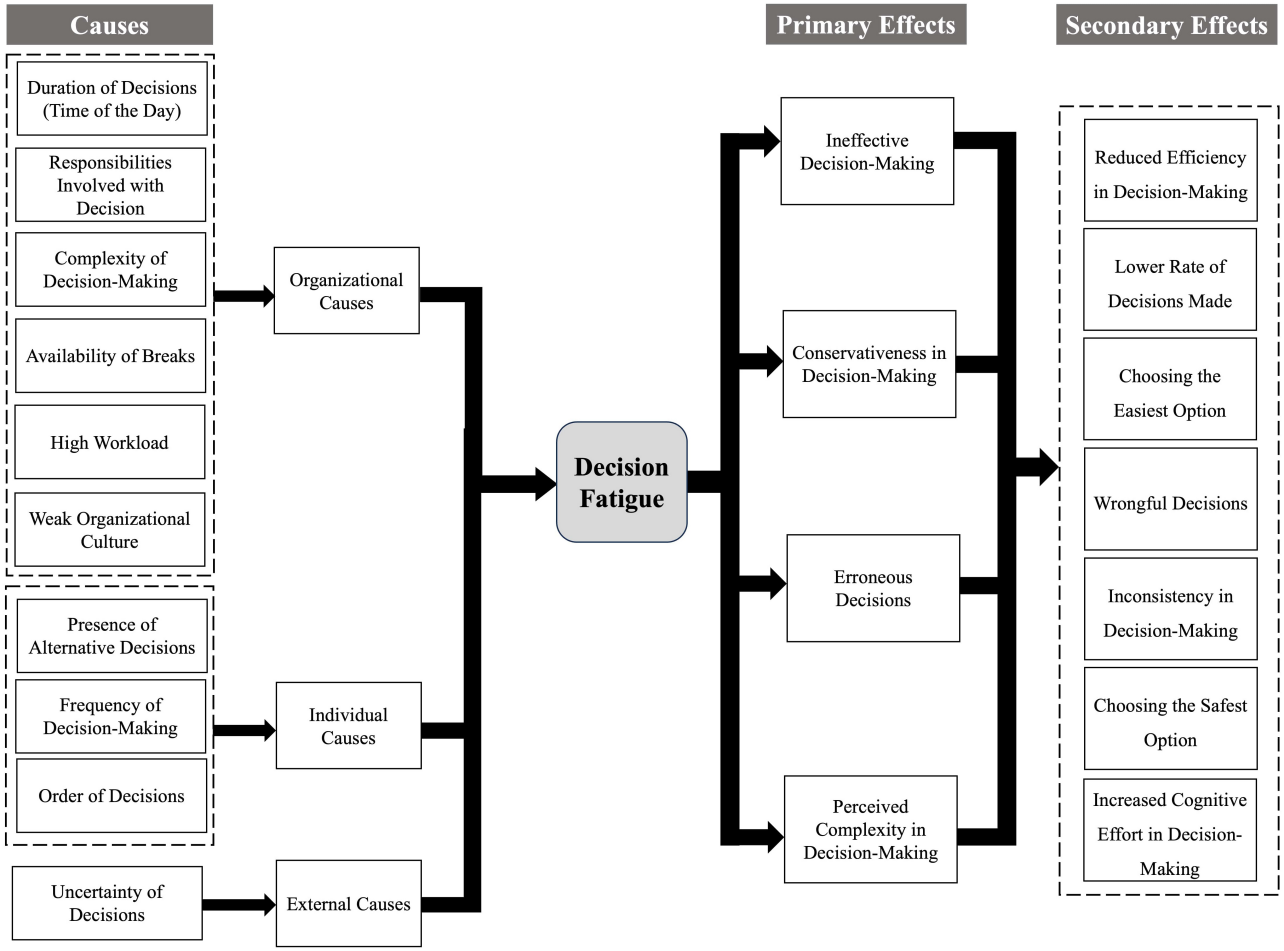
Conceptual framework of the causes and effects of decision fatigue. The framework includes three categories of causes (organizational, individual, and external) encompassing ten contributing factors, which lead to decision fatigue at the center of the model. Four primary effects and seven secondary effects are depicted, showing the progressive impact of decision fatigue on decision-making performance and cognitive effort.

The causes and effects found in the previous sections aided in developing a generalizable conceptual framework presenting the causes and effects of decision fatigue, which is shown in Figure 2. Based on the effects discussed in the 23 articles, we have found seven secondary effects of decision fatigue. An individual is generally affected by one or more of the primary effects once they are subjected to decision fatigue. These primary effects give rise to secondary effects, further deteriorating their decision-making. In our proposed framework in Figure 2, we have highlighted the following secondary effects of decision fatigue, (1) reduced efficiency in decision-making (Allan et al., 2019; Torres and Williams, 2022; Treasure et al., 2022), (2) lower rate of decisions made (Zheng et al., 2020; Hunt et al., 2021; Trinh et al., 2021), (3) choosing the easiest option (Baer and Schnall, 2021; Natal and Saltzman, 2022), (4) wrongful decisions (Mullette-Gillman et al., 2015; Pignatiello and Hickman, 2019), (5) inconsistency in decision-making (Olsen et al., 2017; Persson et al., 2019), (6) choosing the safest option (Torres and Williams, 2022), (7) increased cognitive effort in decision-making (Ma et al., 2021; Shroff and Vamvourellis, 2022).

## 4 Discussion

This integrative review synthesized the findings from 23 articles to develop a conceptual framework for exploring the causes and effects of decision fatigue across multiple domains. This framework contributes toward improving the decision-making process by offering a generalized understanding of decision fatigue. Moreover, our findings contribute to the theoretical understanding of occupational decision-making by offering a multi-domain conceptual framework that explores the cognitive mechanisms of decision fatigue. Previously, Almonroeder et al. (2020) conducted a systematic review to investigate the effect of physical fatigue on athletes' decision-making in high-intensity sports activities. Although they had analyzed articles that discuss cognitive processing during fatigue and stress, their focus was confined to a more specialized area compared to our approach of involving a broader investigation of decision fatigue in diverse domains. Similarly, Perry et al. (2025) conducted a systematic review on decision fatigue, exploring its causes, effects, and implications for decision-making in healthcare. While their focus was exclusively on clinical decision-making, our study takes a broader and multi-domain approach, incorporating healthcare, finance, judiciary, and other workplace domains. We have broadened the scope further to develop a conceptual framework summarizing documented associations and effects of decision fatigue reported across included studies. The identified causes reported in this review should be interpreted as associations rather than confirmed causal relationships with decision fatigue. These findings refine rather than replace the existing theoretical concept of decision fatigue which focused on specific domains. For example, decision fatigue in nursing is reported to be caused by personal and organizational drivers such as fatigue, staffing shortages, and workload (Goudarzian et al., 2025). Similarly, systematic reviews by Grignoli et al. (2025) and Maier et al. (2025) identified sector-specific causes of decision fatigue in healthcare. Our study develops a multi-domain conceptual framework that builds on prior domain-specific studies (Zheng et al., 2020; Treasure et al., 2022; Fernández-Miranda et al., 2023) and supports preliminary generalization while acknowledging that healthcare studies dominate the current literature. However, knowledge of domain-specific causes of decision fatigue can help decision-makers in other domains develop awareness of the possibilities for those causes of decision fatigue in their own domains. Thus, this framework should be regarded as boundary-sensitive and subject to further validation in non-clinical environments. Knowledge of all possible causes of decision fatigue can help optimize the decision-making process. Furthermore, understanding the causes of decision fatigue can help develop interventions to overcome or prevent them from occurring in diverse organizational domains. These insights are crucial for organizational decision-makers to optimize decision-making processes and enhance the quality of their decisions.

We further synthesized the articles to determine the effects of decision fatigue and its consequences on decision-making effectiveness. Decision fatigue reduces decision-making efficiency by depleting cognitive resources, making it difficult for individuals to react appropriately and make optimal decisions. Our findings suggest that decision fatigue deteriorates decision-making quality and efficiency, supporting the results of prior studies by Moorhouse (2020), and Pignatiello et al. (2020), which also highlighted the impact of cognitive depletion on decision quality. Our review extends these findings by categorizing the effects as primary and secondary effects, with secondary effects stemming from primary effects. The categorizations of primary and secondary effects are represented as generalized, immediate and long-term effects, rather than a definitive sequential relationship among the categories. Furthermore, decision fatigue can reduce the ability to make quick and frequent decisions. This inability prompts individuals to be more selective or conservative in their decision-making to manage the depletion of mental resources, resulting in a lower decision-making rate. This tendency to opt for the safest option can be a way to avoid the cognitive load associated with more complex decisions under fatigued conditions. Individuals might have to put extra effort into overcoming fatigue and reach a decision while trading off the quality of their choices. These results corroborate the findings of Mullette-Gillman et al. (2015), where it was shown that decision fatigue causes higher levels of effort during cognitively taxing activities. Understanding these severe effects of decision fatigue on individual and organizational performance can help develop strategies to measure decision fatigue and find remedies to control it objectively.

The framework developed through this integrative review in various organizational domains helps generalize the causes and effects of decision fatigue. This study contributes to occupational psychology by identifying how decision fatigue occurs from high workload, organizational demands, and cognitive load, and how it affects organizational performance. Our findings can also help develop targeted interventions to mitigate the effects of decision fatigue and improve overall decision-making and improve the occupational psychology of individuals. For example, one of the causes of decision fatigue mentioned in our study is the time of the day, since decision fatigue increases as the day or shift progresses. To control the effects of this factor, organizations can allocate cognitively demanding tasks to periods when employees are starting their shifts or immediately following a break. Similarly, organizations can also benefit from the knowledge of the detrimental effects of decision fatigue. Decision fatigue decreases the quality of decision-making but also increases the risk of stress and burnout for the decision-makers. Making wrongful decisions and dealing with their after-effects can increase mental stress on individuals and can even result in post-traumatic stress disorder (PTSD) arising from poor decisions and their consequences (Ehlers and Clark, 2000; Wang et al., 2023). These challenges become even more daunting in high-risk and crisis scenarios. Reale et al. (2023) showed that decision-making becomes increasingly difficult in high-risk domains, such as healthcare, aviation, and the military, highlighting the role of stress and time pressure in undermining decision-making quality and emphasized adopting effective strategies to manage these situations. By integrating evidence across multiple domains, our study advances the theoretical and practical implications on how decision fatigue influences human decision-making within high-stakes environments. Finally, our study can further contribute to the prevalent research on decision-making under crisis by providing insights into decision fatigue and its effects while providing practical implications for improving organizational cognitive performance. While most of the evidence comes from healthcare settings, the findings can inform decision-making strategies to mitigate decision fatigue in high-risk domains like aviation, firefighting, and other emergency response sectors.

Despite the comprehensive nature of our integrative review, which unveils the causes and consequences of decision fatigue, certain limitations prevail. There is a lack of research that directly explores the causes and effects of decision fatigue. This created a challenge in selecting the appropriate databases for literature searches and finding research articles that explicitly discuss decision fatigue. Moreover, the included studies are mostly domain-specific, as past research has primarily focused on studying the causes and/or effects of decision fatigue in a specific domain, such as healthcare. As a result, the findings of this review are heavily concentrated in healthcare, which may restrict the generalizability of the proposed framework to other occupational domains. Although our intent was to capture cross-domain patterns, some reported factors and outcomes may be specific to particular professional roles or work environments. Therefore, the multi-domain conceptual framework should be validated in other domains in future research. As a next step, we aim to validate the decision fatigue framework by conducting a Delphi study with domain-specific decision-makers and studying how the conceptual framework can be applied or modified across domains. Furthermore, **t**he urgency of domain-specific research on decision fatigue cannot be overstated. By focusing on these causes and effects, we can develop more effective mitigation strategies tailored to the domain's unique challenges. Expanding the focus of research to include a more diverse range of professionals would help generalize the findings and ensure that interventions are effective across different domains and professionals. Additionally, future research can be designed to objectively measure and quantify decision fatigue. This rigorous approach would enable prioritizing the causes in terms of severity to decide which causes must be controlled first, leading to more targeted interventions.

## 5 Conclusion

Our integrative review and conceptual framework contribute to the growing body of knowledge on decision fatigue. By synthesizing diverse findings, we provide a foundation for future research and offer practical insights for professionals seeking to optimize decision-making processes in various contexts. To our knowledge, the present review is unique in developing a framework for the causes and effects of decision fatigue and addresses decision-making across multiple domains. A systematic literature review of such nature can pave the way for future researchers to find ways to mitigate decision fatigue among professionals, thereby enhancing decision-making efficiency and employee well-being. Future research can test how the proposed framework works under uncertain emergencies or crises. The overall implication of this research lies in its potential to enhance the decision-making processes, optimize outcomes, and contribute to increasing the accuracy and quality of the process under the influence of decision fatigue.

## Data Availability

The original contributions presented in the study are included in the article/supplementary material, further inquiries can be directed to the corresponding author.

## Appendix A: Search strings

**Appendix A.1:** Search strings with results.

**Database**	**Search string**	**Results**
APA PsycInfo	noft(Decision Fatigue) AND noft(Decision Making OR Crisis OR Emergency)	542
Scopus	{Decision Fatigue} AND (“decision making” AND crisis*)	47
PubMed	(“decision fatigue”[Title/Abstract] OR “ego depletion”[Title/Abstract]) AND (“decision making”[MeSH Terms] OR “decision making”[Title/Abstract] OR “cognitive performance”[Title/Abstract]) AND (“crisis”[Title/Abstract] OR “emergency”[Title/Abstract] OR “stressful situation”[Title/Abstract]) AND (“2001/01/01”[Date - Publication]: “2023/12/31”[Date - Publication]) AND (english[Language])	435
